# Assessment of Knowledge on Insulin Administration Among Diabetes Mellitus Patients in Kathmandu Valley

**DOI:** 10.1002/puh2.70164

**Published:** 2025-11-24

**Authors:** Swekriti Puri, Prabesh Baniya

**Affiliations:** ^1^ JF Institute of Health Sciences Tribhuvan University Kathmandu Nepal; ^2^ Department of Pharmacy Kathmandu University Dhulikhel Nepal

**Keywords:** diabetes mellitus, health education, hyperglycemia, insulin administration, knowledge, practice

## Abstract

**Background:**

Diabetes mellitus (DM) is a chronic metabolic disease resulting from insulin deficiency, leading to hyperglycemia. Effective management, particularly for insulin‐dependent patients, relies heavily on correct insulin administration. Assessing patient knowledge regarding this is crucial for identifying educational needs.

**Objectives:**

This study aimed to assess the level of knowledge on insulin administration among DM patients attending Kathmandu Diagnostic Center, Lalitpur.

**Methods:**

A descriptive, cross‐sectional study was conducted from February 2, 2024, to April 2, 2024. A total of 187 DM patients were included. Data were collected using a semi‐structured questionnaire covering various aspects of insulin administration and analyzed using Statistical Package for Social Science (SPSS) version 23.

**Results:**

The study found that 84% of participants had adequate knowledge of insulin administration, though gaps existed in understanding insulin types and postinjection care. Most patients demonstrated good adherence to injection techniques such as priming and aseptic practices, but fewer consistently checked insulin expiry or practiced proper needle disposal. Knowledge and practice were significantly associated with diabetes duration, insulin therapy length, and age, whereas the type of delivery device showed no significant impact.

**Conclusions and Recommendations:**

This study demonstrates that although the majority of patients with DM in Nepal possess adequate knowledge of insulin administration, critical gaps remain, particularly in understanding insulin complications, types, and comprehensive postinjection care. Knowledge positively correlates with correct insulin administration practices, underscoring the importance of patient education. Moreover, longer disease duration, extended insulin therapy, and older age are significantly associated with better knowledge and adherence, whereas the type of insulin delivery device does not influence these factors. These findings highlight the urgent need for targeted, age‐ and experience‐tailored educational interventions to improve insulin management skills and self‐care practices. Improving patient literacy and technique can contribute substantially to optimizing glycemic control and reducing diabetes‐related complications in this population.

## Graphical abstract

This study assessed insulin administration knowledge among diabetes patients in Kathmandu Valley, revealing that although most demonstrated adequate knowledge, critical gaps remain in expiry checks, insulin types, and postinjection care. Targeted education is essential to improve safe insulin practices and reduce diabetes‐related complications.

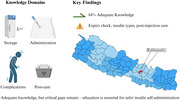

## 1 Introduction

Diabetes mellitus (DM) is a severe, chronic metabolic disease due to insulin deficiency or ineffective use, affecting 425 million people globally, primarily in low/middle‐income countries. In Nepal, DM prevalence is estimated to be between 4.1% and 9.5% [1]. Effective long‐term management and proper utilization of insulin critically depend on the patient's ability to correctly and consistently self‐administer their prescribed doses at home [2].

Individuals with DM face an increased risk of cardiovascular, peripheral vascular, and cerebrovascular diseases. Lifestyle modification, encompassing key aspects such as nutritional management, regular physical activity, blood glucose monitoring, and patient education, plays a vital role in the effective management of this condition [3, 4]. Proper insulin injection practice is essential for better diabetic control [5]. Diabetes is considered one of the sixth leading causes of mortality in the world. It is estimated to become the seventh leading cause of death by 2030. Health literacy plays a significant role in self‐care, adherence to medication, and clinical outcomes in diseased individuals [6, 7]. The storage conditions of insulin can also affect its potency. It can lead to glycemic variability and subsequently compromised short‐ and long‐term complications. Such aftermaths include injection site‐related pain, bruising, allergy, infection, and lipodystrophy [8, 9]. The global prevalence of diabetes has rapidly increased over the past decade, and close to 600 million people are predicted to develop diabetes by 2035 globally [10]. In the study regarding insulin storage, more than half of them store opened insulin in the refrigerator. Appropriate injection site rotation was reported by nearly one‐third of the participants [11]. The study carried out in China found that about 2.99% of patients reported having an infection at the injection sites. Most patients (72.55%) occasionally felt pain when injecting insulin [12]. Effective glycemic control hinges on various aspects of insulin injection practices, including insulin storage, potential mixing when using syringes, and selecting and rotating injection sites [13]. The American Diabetes Association (ADA) has indicated that effective self‐management is achieved by a structured and individualized plan based on a person's needs. In a tertiary care center, around 32.33% of the patients were disposing of the used syringes and vials appropriately [14]. The findings indicated that 72.33% (*n* = 217) displayed a moderate level of knowledge, whereas 27.66% (*n* = 83) demonstrated an adequate level of expertise. In the study carried out in Sri Lanka, 90 participants (25%; CI: 20.7%–29.7%) were identified as suffering from skin changes, followed by 15.3% (95%; CI: 11.8–19.2) with persistent swelling [14, 15], and among people with T2DM, 126 individuals were identified with wrong injection practices. The site of injection was incorrect in 20.17% of the patients [16]. Studies in our neighboring countries, India and China, also showed a significant gap between the insulin administration guidelines and insulin injection technique [8, 17, 18]. Moreover, it has also been reported that faulty injection technique can cause insulin allergy [18, 19]. Considering this scenario, knowledge regarding insulin injection might be poor among Nepalese patients with DM. Therefore, it seemed rational to assess insulin injection practice among diabetes patients.

## 2 Materials and Methods

### 2.1 Study Design

A descriptive, cross‐sectional study design was employed for this research, involving 187 participants with DM. The study was conducted at the Kathmandu Diagnostic Center from June 9, 2023, to June 21, 2023. Data were collected using a semi‐structured questionnaire designed to assess participants’ knowledge of insulin administration and their self‐reported insulin injection practices. Ethical approval for the study was obtained from the Institutional Review Committee of CIST College, Nepal. Permission to conduct the research was also secured from the administrative department of the Kathmandu Diagnostic Center. The study was conducted in adherence with established research ethics principles.

### 2.2 Study Population and Inclusion Criteria

The study population comprised patients with a confirmed diagnosis of Type 1 or Type 2 DM who were currently receiving insulin therapy, either as a primary treatment or as an adjunct to oral hypoglycemic agents. Patients with Type 1 diabetes were typically insulin‐dependent from the time of diagnosis, whereas those with Type 2 diabetes were included if their glycemic control was deemed inadequate on oral agents alone, necessitating insulin therapy. No participants were excluded on the basis of diabetes type, as the study aimed to evaluate knowledge of insulin administration comprehensively across both groups.

The methodology did not explicitly differentiate between Types 1 and 2 DM; rather, the inclusion criterion was insulin use regardless of diabetes classification. Specifically, both patient groups were included under the premise of insulin therapy as either primary or adjunctive treatment:

Type 1 diabetes: Patients are primarily reliant on insulin from diagnosis for survival.
Type 2 diabetes: Patients using insulin as an adjunct or alternative to oral hypoglycemic agents.

This inclusive approach ensured that the study captured a representative sample of insulin users, facilitating a comprehensive assessment of insulin administration knowledge. Patient diabetes type (Type 1 vs. Type 2) was recorded during data collection to enable subgroup analyses and comparisons.

### 2.3 Sample Size

Determination: Sample size was 187 DM patients. The sample size is calculated by using the Cochrane formula.

Calculation

$$N=\frac{Z^{2}Q}{D^{2}}$$

where *N* is the sample size, *Z* is 1.96 (taking a confidence interval of 95% into account), and *P* is the prevalence = 8.5% (8.5/100) = 0.085.

Very little is known about the burden of diabetes and prediabetes, treatment, and control across provinces of Nepal. Current evidence shows the prevalence rate of DM is 8.5% in Nepal [20].

Q=1−P(1−0.085)=0.915

$$D=\;\mathrm{margin}\mathrm{of}\mathrm{error}\left(\mathrm{precision}\right)=4\%=\;0.04$$

$$N={\left(1.96\right)}^{2}\times \;0.085\;\;\times \;0.915/{\left(0.04\right)}^{2}=186.73$$

Thus, a sample size of 187 will be selected for the study.

### 2.4 Sampling Technique

A convenience sampling technique was used to recruit participants from the Kathmandu Diagnostic Center during the study period.

### 2.5 Data Collection Instrument and Procedures

Data were collected using a semi‐structured questionnaire developed after reviewing prior studies on insulin administration knowledge and practices. The tool was designed to assess four main domains:

1. Knowledge of diabetes and insulin—awareness of the disease, insulin indications, and related complications.
2. Knowledge of insulin storage—proper handling, refrigeration, and precautions after opening vials/pens.
3. Knowledge of insulin administration technique—injection sites, site rotation, priming, aseptic techniques, and injection hold time.
4. Knowledge of complications and self‐care—recognition of hypoglycemia, safe disposal of injection devices, and postinjection practices.

To ensure validity, the questionnaire was reviewed by three subject matter experts in diabetes care. It was initially prepared in English, translated into Nepali for local use, and back‐translated to English to maintain accuracy and conceptual equivalence. A pilot test was conducted on 10 patients to refine clarity and cultural relevance. Modifications were made accordingly before implementation. Reliability testing yielded a Cronbach's alpha of 0.72, indicating acceptable internal consistency.

Written informed consent was obtained from all participants prior to data collection. Patients who declined consent or were unable to respond were excluded.

### 2.6 Study Variables: The Study Variables Are

#### 2.6.1 Sociodemographic Independent Variables

– Age
– Gender
– Ethnicity/Caste
– Religion

#### 2.6.2 Knowledge Variables

– Knowledge regarding disease
– Knowledge regarding storage
– Knowledge regarding the technique of administration

### 2.7 Data Processing and Analysis

The collected data were entered, checked, and edited manually, and data analysis was done as per the objectives of the study. Analysis was performed on the Statistical Package for Social Sciences (SPSS) software version 23, acquired by IBM.

## 3 Results

### 3.1 Sociodemographic Characteristics of Participants

A total of 187 individuals diagnosed with DM participated in this descriptive, cross‐sectional study conducted in Lalitpur District. The majority of participants (52.9%) were between 50 and 70 years of age, followed by 23.0% in the 30–50 years group, 21.4% aged over 70 years, and only 2.7% were under the age of 30. The gender distribution showed a predominance of females (57.2%) compared to males (42.8%). Regarding marital status, 96.3% of participants were married, whereas only 3.7% were unmarried, reflecting the typical age distribution and social structure of individuals managing long‐term conditions such as diabetes. In terms of educational attainment, the largest group (41.7%) had completed secondary‐level education, followed by 34.2% with primary education, 15.5% with higher secondary, and only 8.6% with graduate‐level or higher education. Occupationally, the most frequent response fell under the “others” category (39.6%), indicating a diversity of informal or unclassified jobs. This was followed by employees (25.7%), agricultural workers (17.6%), business (11.2%), service sector (3.2%), and laborers (2.7%). All participants in this study identified as Hindu (100%), which is consistent with the religious demographic of the region. Most respondents (86.1%) resided in urban areas, whereas only 13.9% were from rural communities, reflecting the urban setting of the study site. When asked about family history of diabetes, a significant proportion (58.8%) reported a first‐degree relative with the condition, 40.6% reported a second‐degree relative, and only 0.5% had no known family history (Table 1).

**TABLE 1:** Demographic details of participants.

Demographic characteristics	No. of participants *N* = 187	Percentage
**Age**		
<30 years	5	2.7
30–50 years	43	23
50–70 years	99	52.9
>70 years	40	21.4
**Sex**		
Female	107	57.2
Male	80	42.8
**Marital status**		
Unmarried	7	3.7
Married	180	96.3
**Occupational status**		
Employee	48	25.7
Agriculture	33	17.6
Labor	5	2.7
Business	21	11.2
Service	6	3.2
**Religion**		
Hindu	187	100
**Educational status**		
Primary‐level	64	34.2
Secondary‐level	78	41.7
Higher secondary	29	15.5
Graduation and above	16	8.6
**Family history**		
First‐degree relative	110	58.8
Second‐degree relative	76	40.6
No relation	1	0.5
**Residential area**		
Urban	161	86.1
Rural	26	13.9

### 3.2 Disease‐Related Characteristics of Participants

Among the 187 participants, the majority (54.0%) had been diagnosed with diabetes for 10–20 years, indicating a long‐standing disease burden within the study population. A further 28.9% had been living with diabetes for 1–10 years, whereas 15.0% had diabetes for over 20 years. Only 1.6% had a disease duration of less than 1 year, and 0.5% reported no confirmed diagnosis, which may reflect an early stage or undiagnosed condition under insulin treatment.

In terms of insulin therapy duration, 48.7% of participants had been on insulin for 1–5 years, whereas 41.7% had been using insulin for more than 5 years, suggesting a relatively high prevalence of long‐term insulin dependence. Only 9.6% had initiated insulin therapy within the last year, which may represent either recently diagnosed individuals or transitions from oral hypoglycemics. When asked about insulin delivery methods, pen injectors were the most commonly used device (71.7%), whereas 28.3% used traditional insulin syringes. This reflects a growing preference for user‐friendly and portable insulin delivery technologies, which may influence adherence and technique‐related outcomes (Table 2).

**TABLE 2:** Disease characteristics.

Disease characteristics	Frequency	Percentage
**For how long have you had diabetes**		
<1 year	3	1.6
1–10 years	54	28.9
10–20 years	101	54.0
>20 years	28	15
None	1	0.5
**For how long have you been on insulin therapy**		
3 months–1 year	18	9.6
1–5 years	91	48.7
>5 years	78	41.7
**Device used**		
Pen injector	134	71.7
Insulin syringe	53	28.3

### 3.3 Knowledge Assessment on Diabetes and Insulin Use

The assessment of participants’ knowledge revealed considerable variation across different domains. The mean knowledge score for diabetes was 74.63% (SD = 13.58), ranging from 44.44% to 88.89%, suggesting a relatively strong foundational understanding. However, knowledge on insulin storage was substantially lower, with a mean of 51.12% (SD = 15.81), and ranged from 20.00% to 100.00%, indicating inconsistencies and potential gaps in handling insulin properly. Knowledge of insulin administration averaged 54.55% (SD = 9.46), suggesting moderate familiarity with procedures such as injection techniques. Awareness of diabetes‐related complications was the poorest among domains, with a mean score of 33.16% (SD = 12.84), underscoring a critical need for improved education on long‐term risks and adverse outcomes of poor glycemic control. When computed across all assessed domains, the overall mean knowledge score was 55.36% (SD = 5.55), reflecting moderate competency among participants. Classification based on the overall knowledge score revealed that 84.0% of participants demonstrated adequate knowledge, whereas 16.0% were classified as having inadequate knowledge (Table 3).

**TABLE 3:** Knowledge overall percentage regarding diabetes and insulin.

Descriptive statistics					
	*N*	Minimum	Maximum	Mean	Std. deviation
Knowledge_diabetes_per	187	44.44	88.89	74.6286	13.58334
Knowledge_storage_per	187	20.00	100.00	51.1230	15.80530
Knowledge_administration_percentage	187	20.00	90.00	54.5455	9.45742
Knowledge_complication_per	187	0.00	75.00	33.1551	12.84492
Knowledge_overall_per	187	37.93	65.52	55.3568	5.54789
Valid *N* (listwise)	187				

		Frequency	Percent	Valid percent	Cumulative percent
Valid	Inadequate	30	16.0	16.0	16.0
	Adequate	157	84.0	84.0	100.0
	Total	187	100.0	100.0	

### 3.4 Knowledge Regarding Insulin Administration Practice

The results provide valuable insights into the knowledge and practices of patients regarding insulin administration, highlighting both strengths and areas in need of improvement. With respect to safety practices, a little over half of the respondents (58.3%) reported consistently checking the expiry date of insulin before use, whereas a notable proportion (41.7%) failed to do so. This finding is clinically significant, as the use of expired insulin can lead to reduced potency, poor glycemic control, and potential health risks. Knowledge regarding insulin formulations also appeared limited; although the majority of participants (73.8%) were aware of long‐acting insulin, awareness of intermediate‐acting (20.3%) and fast‐acting insulin (5.9%) was comparatively low, suggesting a gap in patient education concerning the pharmacokinetics and therapeutic uses of different insulin types. Encouragingly, adherence to correct injection techniques was high, as almost all participants primed their insulin pens (98.9%), cleaned the injection site prior to administration (93.6%), and reported following aseptic techniques (96.3%), all of which are essential for ensuring proper drug delivery and minimizing the risk of infection. Similarly, the majority (86.1%) adhered to the recommended 10‐s hold time following injection, thereby ensuring full dose delivery and reducing insulin leakage. However, practices related to postinjection care were less optimal. Although nearly half of the respondents (48.7%) reported proper needle disposal and 41.7% monitored blood glucose after injection, only 9.6% engaged in cleaning the site post‐administration, reflecting inconsistencies in the completion of the full care process. Taken together, these findings demonstrate that although patients generally possess strong skills in the technical aspects of insulin injection, deficiencies persist in knowledge of insulin types, medication safety checks, and comprehensive postinjection care. This underscores the importance of targeted educational interventions to enhance patient understanding of insulin pharmacology, reinforce safe handling practices, and promote holistic self‐care behaviors for optimal diabetes management (Table 4).

**TABLE 4:** Participants’ knowledge on insulin administration practice.

Variables	Frequency	Percent	Valid percent	Cumulative percent
1. Checking insulin expiry				
a. Checked	109	58.3	58.3	58.3
b. Not checked	78	41.7	41.7	100.0
2. Understanding different insulin types				
a. Fast‐acting	11	5.9	5.9	5.9
b. Intermediate acting	38	20.3	20.3	26.2
c. Long acting	138	73.8	73.8	67.9
3. Priming insulin pen				
a. Yes	185	98.9	98.9	98.9
b. No	2	1.1	1.1	100.0
4. Cleaning the injection site				
a. Yes	175	93.6	93.6	93.6
b. No	12	6.4	6.4	100.0
5. Follow aseptic techniques				
a. Yes	180	96.3	96.3	96.3
b. No	7	3.7	3.7	100.0
6. Injection hold time				
a. 10 s	161	86.1	86.1	86.1
b. More than 10 s	26	13.9	13.9	100.0
7.Postinjection care				
a. Cleaning the site of injection	18	9.6	9.6	9.6
b. Dispose needles	91	48.7	48.7	58.3
c. Blood sugar monitoring	78	41.7	41.7	100.0

### 3.5 Practical Skills Related to Insulin Administration

An observational assessment was conducted to evaluate participants’ practical skills in self‐administering insulin using a structured checklist. All participants (100%) correctly identified appropriate insulin injection sites, suggesting strong foundational knowledge in this domain. Regarding injection site rotation, a crucial technique to prevent lipohypertrophy and ensure consistent insulin absorption, 96.8% demonstrated correct rotation, whereas 2.7% skipped rotation, and 0.5% performed it incorrectly. However, notable gaps were observed in other key procedural aspects. Only 36.4% of participants correctly shook NPH insulin prior to injection, an essential step to ensure proper resuspension of the insulin. A significant 61.0% skipped this step, and 2.7% performed it incorrectly, indicating a potential area for intervention.

Concerning the technique of pinching the skin and injecting at a 45° angle, 71.1% executed the step correctly, whereas 24.6% skipped it, and 4.3% performed it incorrectly, which could compromise insulin delivery efficacy.

Alarmingly, only 8.0% of participants correctly demonstrated how to draw insulin from a vial, whereas 88.8% skipped this step, and 3.2% performed it incorrectly, underscoring a substantial deficit in this fundamental competency. This finding reflects a critical need for enhanced patient education and hands‐on training regarding insulin preparation techniques (Table 5).

**TABLE 5:** Observation checklist of patient's skill related to self‐insulin administration.

Observation of insulin practice	Frequency	Percentage
**Showed injection sites**		
Correct	187	100
**Injection site rotations**		
Correct	181	96.8
Incorrect	1	0.5
Skipped	5	2.7
**Showed to shake NPH**		
Correct	68	36.4
Incorrect	5	2.7
Skipped	114	61
**Showed how to pinch skin and inject with a 45° angle**		
Correct	133	71.1
Incorrect	8	4.3
Skipped	46	24.6
**Showed how to draw insulin from the vial**		
Correct	15	8
Incorrect	6	3.2
Skipped	166	88.8

### 3.6 Association Between Knowledge and Insulin Administration Practices

Table 5 indicates a statistically significant association between participants’ knowledge levels and their insulin administration practices (*χ* ^2^ = 6.250, *p* = 0.012). Among participants with inadequate knowledge, only 56.7% (17 out of 30) demonstrated good practice, whereas 43.3% (13 out of 30) exhibited poor practice. In contrast, 78.3% (123 out of 157) of participants with adequate knowledge demonstrated good insulin administration practices, with only 21.7% (34 out of 157) categorized as having poor practice. These results suggest that increased knowledge about diabetes and insulin administration positively correlates with proper injection technique, reinforcing the critical role of targeted patient education and training programs (Table 6).

**TABLE 6:** Association between knowledge and insulin administration practices.

	Poor	Good	Value	*p* value
Inadequate	13	17	6.250	0.012
Adequate	34	123		

### 3.7 Knowledge vs. Disease Characteristics and Age

The relationship between patients’ insulin administration knowledge and various disease and demographic factors was assessed using *p* values to determine statistical significance. Checking insulin expiry was significantly associated with duration of diabetes (*p* < 0.001), insulin therapy duration (*p* = 0.041), and age (*p* = 0.020), but not with the type of insulin delivery device (*p* = 0.062). Similarly, knowledge of different insulin types demonstrated significant associations with duration of diabetes (*p* = 0.004), insulin therapy duration (*p* = 0.038), and age (*p* = 0.032), whereas the device used showed no significant effect (*p* = 0.081). Priming the insulin pen was significantly influenced by duration of diabetes (*p* < 0.001), insulin therapy duration (*p* < 0.001), and age (*p* = 0.028), but not by the device type (*p* = 0.054). Cleaning the injection site and adherence to aseptic techniques were both significantly associated with duration of diabetes (*p* < 0.001 for both), insulin therapy duration (*p* = 0.016 and *p* = 0.022, respectively), and age (*p* = 0.039 for cleaning; *p* = 0.081 for aseptic technique), whereas the device used did not show a significant effect in either case. Injection hold time was significantly related to duration of diabetes (*p* = 0.033), insulin therapy duration (*p* = 0.049), and age (*p* = 0.041), but not to device type (*p* = 0.089). Finally, postinjection care practices were significantly associated with duration of diabetes (*p* = 0.025), insulin therapy duration (*p* = 0.038), and age (*p* = 0.029), whereas the type of device again did not have a significant impact (*p* = 0.078). Overall, these findings suggest that patient knowledge and adherence to insulin administration practices are largely influenced by the length of their diabetes history, duration of insulin therapy, and age, whereas the type of insulin delivery device does not appear to play a major role (Table 7).

**TABLE 7:** Knowledge vs. disease characteristics and age (chi‐square association).

Sl. no.	Knowledge factor	Disease/Demographic factor	*p* value	Significance
1	Checked expiry	Duration of diabetes	<0.001	Significant
		Insulin therapy duration	0.041	Significant
		Device used	0.062	Not significant
		Age	0.020	Significant
2	Insulin type	Duration of diabetes	0.004	Significant
		Insulin therapy duration	0.038	Significant
		Device used	0.081	Not significant
		Age	0.032	Significant
3	Priming	Duration of diabetes	<0.001	Significant
		Insulin therapy duration	<0.001	Significant
		Device used	0.054	Not significant
		Age	0.028	Significant
4	Cleaning site	Duration of diabetes	<0.001	Significant
		Insulin therapy duration	0.016	Significant
		Device used	0.072	Not significant
		Age	0.039	Significant
5	Aseptic techniques	Duration of diabetes	<0.001	Significant
		Insulin therapy duration	0.022	Significant
		Device used	0.061	Not significant
		Age	0.081	Significant
6	Injection hold time	Duration of diabetes	0.033	Significant
		Insulin therapy duration	0.049	Significant
		Device used	0.089	Not significant
		Age	0.041	Significant
7	Postinjection care	Duration of diabetes	0.025	Significant
		Insulin therapy duration	0.038	Significant
		Device used	0.078	Not significant
		Age	0.029	Significant

### 3.8 Insulin Injection Sites and Common Hypoglycemic Symptoms

**FIGURE 1:**
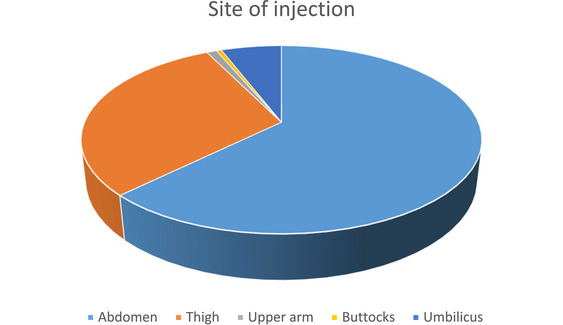
Distribution of insulin injection sites (pie chart).

The selection of appropriate injection sites is essential for effective insulin absorption and patient comfort. As shown in Figure 1, the abdomen was the most commonly used site for insulin administration, reported by 69% of participants. This preference is consistent with clinical guidelines, as abdominal injections tend to have faster and more predictable absorption rates. Other injection sites included the thigh (33.2%), umbilicus (6.4%), upper arm (1.1%), and buttocks (0.5%). Although some participants reported using more than one site, the dominance of the abdominal region indicates both awareness and adherence to recommended practices (Figure 1).

This pie chart visually represents the frequency distribution of insulin injection sites among study participants.

In addition to injection practices, participants were also assessed for their experiences with hypoglycemic symptoms, as detailed in Table 6. The most commonly reported symptom was sweating, experienced by 36.4% of participants. This was followed by palpitations (17.1%), shaking or trembling (10.2%), and confusion or difficulty in concentration (2.1%). Notably, 33.1% of participants reported experiencing all of the listed symptoms, indicating an awareness of the multifaceted nature of hypoglycemic episodes. Only 1.1% of participants reported no symptoms, suggesting that the vast majority are at least partially aware of the physiological signs of hypoglycemia (Table 8).

**TABLE 8:** Reported hypoglycemic symptoms.

		Frequency	Percent	Valid percent	Cumulative percent
Valid	None	2	1.1	1.1	1.1
	Sweating	68	36.4	36.4	37.4
	Palpitation	32	17.1	17.1	54.5
	Shaking or trembling	19	10.2	10.2	64.7
	Confusion/Difficulty	4	2.1	2.1	66.8
	All of the above	61	33.1	33.1	99.5

## 4 Discussion

Proper insulin injection practice is essential for better diabetic control. This study aims to assess the knowledge of insulin administration among DM patients. The current study revealed that there is adequate knowledge among the patients regarding insulin administration. About 84% had sufficient knowledge, whereas 16% had inadequate knowledge. Diabetes patients had more understanding of diabetes, whereas less knowledge of the complications of insulin.

There is an excellent association between knowledge and practice of insulin. The study showed that as the knowledge increases, the practice of insulin administration also increases.

The current study showed knowledge regarding the complications of insulin injection. The mean value of 33.15 was recorded as complications of insulin injection, and the most common complications of insulin injection among those patients were sweating (71.7%). A similar cross‐sectional study was conducted at Chitwan Medical College Teaching Hospital, Bharatpur, Nepal, to assess the insulin injection practice of patients with diabetes. The current insulin injection technique and complications of insulin injection were recorded. The insulin injection technique of patients and their relatives was inadequate. Thirteen patients (30.2%, *n* = 43) reported complications of insulin injection, and the most common complication among those patients was bruising (10, 76.9%, *n* = 13) [5]. The findings of both studies show different results; the complications among patients were different. The reason behind the differences might be due to the variation of individual patients’ physiology. The study showed that there was a significant gap between the insulin delivery recommendation through an insulin pen and the current insulin injection practice, which highlights the importance of providing information on insulin‐related topics, such as insulin administration and disposal of syringes.

The current study showed that among 187 patients, adequate knowledge (84%), inadequate knowledge (16%), good practice (17), and poor practice (13) for inadequate knowledge and good practice (123), and poor practice (34) for adequate knowledge of self‐administration of insulin among DM patients were found. Similar findings were found in the study conducted in a tertiary care hospital, Vadodara. Among 300 patients, inadequate knowledge (0.33%), moderate knowledge (72%), and adequate knowledge score (27.66%), and poor practice (6.70%), average practice (77%), good practice (12%), and excellent practice score (4%) of self‐administration of insulin among DM patients were found. The results of both studies were comparatively different; the survey done at Vadodara showed that there is less inadequate knowledge in comparison to current research. The reason behind it might be due to the area of study population, it might be due to sample size variation, and it might be due to literacy level. In accordance with the results and findings, there was no correlation between the knowledge score and practice score. This study concluded that there was a lack of knowledge and practice on insulin self‐administration, highlighting the importance of improving the patient's knowledge and practice on the self‐administration of insulin so that the participants can enhance their practice well [21].

The current study revealed that the mean value of knowledge on self‐administration of insulin was 54.54. A similar analysis was done to assess the knowledge regarding self‐administration of insulin injection among DM patients in the diabetic clinic of the primary health center at Al Namas. The study revealed that 60% of the participants have good knowledge regarding self‐administration of insulin injection, 30% have average knowledge regarding self‐administration of insulin injection, and 2 participants (10%) have poor knowledge regarding self‐administration of insulin injection [22]. The findings reveal that patients generally demonstrate good technical knowledge and adherence to safe insulin injection practices, such as priming pens and following aseptic techniques. However, gaps persist in awareness of insulin types, checking insulin expiry, and comprehensive postinjection care like proper needle disposal and site cleaning. These results highlight the need for targeted educational interventions to improve patient understanding and promote safer, more holistic insulin self‐management.

The current study showed that better knowledge was observed concerning timing, that is, (10.2%) inject >2 h before meal, (71.7%) inject 15–30 min before meal, and (18.2%) inject >2 h after dinner. Out of total patients, about 69% inject insulin over the abdomen, (33.2%) inject over the thigh, (1.1%) inject at the upper arm, (0.5%) inject at the buttocks. Out of the total patients, 37.4% injected insulin appropriately at 90° and 60.4% injected insulin appropriately at 45°. A similar study was conducted aimed at assessing knowledge, attitude, and practice towards insulin self‐administration and associated factors among diabetic patients at Zewditu Memorial Hospital (ZMH), Ethiopia. Better knowledge was observed concerning timing (78.4%) and site of insulin injection (89.4%), whereas knowledge on the angle of inclination during insulin administration (43.3%) and complications of insulin therapy (49%) was low. The majority, 177 (72.2%), of the study patients have administered insulin themselves, and only 120 (49.0%) of the patients injected insulin appropriately at 45°. Frequent repetition of the injection site was practiced among 176 (71.8%) patients, and 139 (56.7%) injected insulin before or immediately after food intake. Patients’ knowledge and attitude seem suboptimal, and malpractice of insulin self‐administration was observed. Both studies showed different results; it might be due to differences in the training given by healthcare professionals about the technique of administration of insulin to the patients. Therefore, the gaps between knowledge and practice should be addressed through patient education, and insulin injection should be demonstrated to the patient during each hospital visit [23].

In the study, the education resulted in an increased number of patients who properly remix cloudy insulin, inject insulin into skin, change every time the injection site, use the pen needle only once, prepare a pen for injection, and store insulin [24, 25].

The analysis demonstrated that patient knowledge and adherence to insulin administration practices are significantly associated with duration of diabetes, length of insulin therapy, and age, indicating that more experienced and older patients tend to have better insulin management skills. In contrast, the type of insulin delivery device showed no significant impact on these knowledge and practice factors. These results suggest that educational interventions should prioritize patient experience and demographic characteristics rather than focusing solely on device type to enhance insulin administration outcomes. The study carried out in healthy volunteers showed that glargine presented well‐reproduced flat concentration profiles and no pronounced peaks in activity. NPH, by contrast, showed well‐defined peaks in concentration and glucose disposal, whereas ultralente had highly variable profiles. Within‐subject variability (ANOVA) for insulin exposure over 24 h was 15% for glargine and 19% for NPH, compared with 67% for ultralente (*p *< 0.05, glargine and NPH vs. ultralente) [25].

According to data provided by the American Association of Clinical Endocrinologists, more than two‐thirds of people with type 2 diabetes have suboptimal glucose control (i.e., hemoglobin A1C level > 6.5%). This level of _1c_ control increases the risk of both short‐ and long‐term complications [26].

## 5 Conclusion

This study demonstrates that although the majority of patients with DM in Nepal possess adequate knowledge of insulin administration, critical gaps remain, particularly in understanding insulin complications, types, and comprehensive postinjection care. Knowledge positively correlates with correct insulin administration practices, underscoring the importance of patient education. Moreover, longer disease duration, extended insulin therapy, and older age are significantly associated with better knowledge and adherence, whereas the type of insulin delivery device does not influence these factors. These findings highlight the urgent need for targeted, age‐ and experience‐tailored educational interventions to improve insulin management skills and self‐care practices. Improving patient literacy and technique can contribute substantially to optimizing glycemic control and reducing diabetes‐related complications in this population.

## Author Contributions

**Swekriti Puri:** conceptualization, methodology, investigation, data curation, formal analysis, visualization, project administration, writing—original draft preparation, writing—review and editing. **Prabesh Baniya:** investigation, data curation, supporting analysis, writing—review and editing.

## Conflicts of Interest

The authors declare no conflicts of interest.

## Data Availability

The corresponding author handles the data set and can provide it upon request.
